# Supplementary material for: Effect of intra-articular dexmedetomidine on experimental osteoarthritis in rats
Source: PLoS One. 2021 Jan 12;16(1):e0245194. doi: 10.1371/journal.pone.0245194 (PMC7802966; doi:10.1371/journal.pone.0245194)
Supplement: Attachment — Submitted filename: Letter of Response to Response to Reviewers 2.docx [file pone.0245194.s002.docx]

Letter of Response to Response to Reviewers'.

Dear editor,

On behalf of my team, I would like to thank the you and the reviewers for the time you took to review our paper. Suggestions and corrections were all taken into careful consideration, and pertinent alterations were done in the final manuscript. We appreciate your efforts to improve our writing and welcome further suggestions by the reviewers.

Each commentary made by the editor and reviewers were answered and highlighted in yellow in this letter.

Best regards.

Osvaldo Barbosa

**Response to reviewers:**

The questions and suggestions made by the reviewers were addressed right after each of the items exposed in the document sent by the scientific journal.

**Comments to the Author**

1. If the authors have adequately addressed your comments raised in a previous round of review and you feel that this manuscript is now acceptable for publication, you may indicate that here to bypass the “Comments to the Author” section, enter your conflict of interest statement in the “Confidential to Editor” section, and submit your "Accept" recommendation.

Reviewer #1: (No Response)

2. Is the manuscript technically sound, and do the data support the conclusions?

The manuscript must describe a technically sound piece of scientific research with data that supports the conclusions. Experiments must have been conducted rigorously, with appropriate controls, replication, and sample sizes. The conclusions must be drawn appropriately based on the data presented.

Reviewer #1: Partly

3. Has the statistical analysis been performed appropriately and rigorously?

Reviewer #1: Yes

4. Have the authors made all data underlying the findings in their manuscript fully available?

The [PLOS Data policy](http://www.plosone.org/static/policies.action#sharing) requires authors to make all data underlying the findings described in their manuscript fully available without restriction, with rare exception (please refer to the Data Availability Statement in the manuscript PDF file). The data should be provided as part of the manuscript or its supporting information, or deposited to a public repository. For example, in addition to summary statistics, the data points behind means, medians and variance measures should be available. If there are restrictions on publicly sharing data—e.g. participant privacy or use of data from a third party—those must be specified.

Reviewer #1: Yes

5. Is the manuscript presented in an intelligible fashion and written in standard English?

PLOS ONE does not copyedit accepted manuscripts, so the language in submitted articles must be clear, correct, and unambiguous. Any typographical or grammatical errors should be corrected at revision, so please note any specific errors here.

Reviewer #1: Yes

6. Review Comments to the Author

Please use the space provided to explain your answers to the questions above. You may also include additional comments for the author, including concerns about dual publication, research ethics, or publication ethics. (Please upload your review as an attachment if it exceeds 20,000 characters)

Reviewer #1: In the reviewer opinion, there remain some few questions that are attached:

- Authors should show representative images for histopathological analysis (hematoxylin and eosin);

RESPONSE: Histopathological images were included as suggested by the reviewer.

- The Authors report that DEX produced an improvement in pain. As dexmedetomidine do not have anti-inflammatory effect, how do the Authors explain the effects of dexmedetomidine on pain? Please clarify.

RESPONSE: Clarification for this item was already addressed in the DISCUSSION section.

The effect of dexmedetomidine in the chronic stage of OA was detected in both clinical tests; this effect might be related to both the central and peripheral actions of the drug. The mechanism underlying the intra-articular analgesia caused by dexmedetomidine has not yet been clearly defined; however, it might be similar to the mechanism suggested for clonidine, an α-2 agonist that acts on presynaptic α-2 adrenergic receptors, inhibiting the release of norepinephrine in peripheral afferent nociceptors [31].

- The Authors conclude that intra-articular administration of dexmedetomidine is safe. However, the Authors did not show the histopathological results of the control groups (animals without OA) treated with intra-articular DEX-1 and DEX-2.

RESPONSE: This statement was removed from the conclusion.

In the histological evaluation of the synovial membrane, dexmedetomidine did not produce an anti-inflammatory effect.

7. PLOS authors have the option to publish the peer review history of their article ([what does this mean?](https://journals.plos.org/plosone/s/editorial-and-peer-review-process#loc-peer-review-history)). If published, this will include your full peer review and any attached files.


If you choose “no”, your identity will remain anonymous but your review may still be made public.


**Do you want your identity to be public for this peer review?** For information about this choice, including consent withdrawal, please see our [Privacy Policy](https://www.plos.org/privacy-policy).

Reviewer #1: No
